# Sexual dysfunction in infertile women

**Published:** 2016-02

**Authors:** Zahra Zare, Malihe Amirian, Nahid Golmakani, Reza Mazlom, Mojtaba Laal Ahangar

**Affiliations:** 1 *Faculty of Nursing and Midwifery, * *Sabzevar University of Medical Sciences* *, Sabzevar, Iran.*; 2 *Department of Obstetrics and Gynecology, Faculty of Medicine, Mashhad University of Medical Sciences, Mashhad, Iran.*; 3 *Faculty of Nursing and Midwifery, Mashhad University of Medical Sciences, Mashhad, Iran.*; 4 *Mashhad University of Medical Sciences, Mashhad, Iran.*

**Keywords:** *Sexual problems*, *Fertile*, *Infertile*, *Couples*

## Abstract

**Background:**

Sexual problems have different effects on the life of people by influencing their interpersonal and marital relationships and satisfaction. Relationship between sexual dysfunctions and infertility can be mutual. Sexual dysfunction may cause difficulty conceiving but also attempts to conceive, may cause sexual dysfunction.

**Objective:**

This paper compares sexual dysfunction in fertile and infertile women.

**Materials and Methods:**

In this cross-sectional study, 110 infertile couples referring to Montasarieh Infertility Clinic and 110 fertile couples referring to five healthcare centers in Mashhad were selected by class cluster sampling method. Data collection tools included demographic questionnaire and Glombok-Rust Inventory of Sexual Satisfaction. Data were analyzed through descriptive and analytical statistical methods by SPSS.

**Results:**

There was no significant difference in total score of sexual problems and other dimensions of sexual problems (except infrequency) in fertile 28.9 (15.5) and infertile 29.0 (15.4) women. Fertile women had more infrequency than infertile women (p=0.002).

**Conclusion:**

There was no significant difference between fertile and infertile women in terms of sexual problems. Paying attention to sexual aspects of infertility and presence of programs for training of sexual skills seems necessary for couples.

## Introduction

Sexual problems have different effects on the life of people and affect their interpersonal and marital relationships and satisfaction (1, 2). Female sexual dysfunction is multifactorial condition likely to be associated with numerous anatomical, physiological and psychological factors and likely to affect woman’s self- confidence, quality of life, mental status and relationships (3). The prevalence of sexual problems has increased in recent years. Estant and colleagues have reported that 30-50% of couples suffer from sexual problems (4). One study reported that prevalence of sexual problems in general population of Iran in women and men are 31.5%, and 18.8%, respectively (5).

The crisis of infertility involves experiencing of a lot of losses, some of which are primary or secondary, but all of them are significant. However the loss of the biologic experience of pregnancy and childbirth or the inability to pass on one′s genes to the next generation are the fears with which infertile couples involved (3, 6). Another profound loss involves the damage done to self-esteem which is not easily repaired. This injury affects one′s deepest sense of self, including feelings about masculinity or feminity, and sexuality. It is probably not surprising, therefore, that a couple′s sexual relationship is often the area of their life that is most negatively affected by infertility (3).

With regard in mind of many people, pregnancy is the score of sexual intercourse, when pregnancy does not occur, sexual intercourse is regarded as an ungainly relation in mind of people, and gradually the desire and tendency to have sexual intercourse is decreased (7). Infertility can be the result of sexual problems itself, also having a desirable sexual relationship may increase fertility (8). Relationship between sexual dysfunctions and infertility can be mutual. Sexual dysfunction may cause difficulty conceiving but also attempts to conceive, may cause sexual dysfunction (9). Different researchers have different opinions on the impact of infertility on couple′s relationship. The study of Besharat which was conducted on 45 infertile and 45 fertile women showed that infertile women have more sexual problems than fertile women (10). Also Gulec and colleagues reported that sexual function was not significantly different in fertile and infertile couples (11). The results of various studies showed that the rates and common type of sexual problems in infertility are different (12, 13).

There is a limit of studies which investigate the effects of infertility on sexual dysfunction in Iran. Merghati *et al* investigated sexual behaviors in Rafsanjan. They revealed women’s sexual self- understandings and their sexual behaviors are strongly determined by “androcentricity”, being relevant both to sexuality education and reproductive health (14).

This paper compares women sexual dysfunction in fertile and infertile women. Given essential role of producing and raising children in Iranian families and its socio-cultural aspects, and due to importance of sexual issues in infertile couples, and since there is little research in this area, we did this survey. Specific value of this study is performing the study on infertile women and also having a control group (fertile women) for comparison.

## Materials and methods

This cross-sectional study was performed on 220 women including 110 infertile women referred to the Montaserieh Infertility Center and 110 fertile women referred to the selected health centers of Mashhad. Study was approved by the Ethics Committee of Mashhad University of Medical Sciences.

To determine the sample size, due to the lack of similar studies, the results of the pilot study performed on 30 cases in two groups of infertile and fertile couples and the formula of comparing means with confidence coefficient of 95% and test power of 80% were used. Calculation of 7 sub-scales of; infrequency, non-communication, dissatisfaction, avoidance, non-sensuality, vaginismus, and anorgasmia was separately performed. 

The highest calculation was related to the sub-scale of non-relationship with 99 cases in each group. For greater certainty, 110 cases in each group and a total of 220 women were studied. Sampling of fertile couples was performed as class-cluster sampling. So, health center was randomly selected among Mashhad quintet health centers at first, in each selected center, in proportion to the volume and number of clients per day, 10 days of month were selected by Lottery Choice and eligible clients in those days were selected as samples. In Montaserieh Infertility Center, sampling was performed as easy sampling because the different classes of people were referred.

Inclusion criteria included Iranian, age of 18-45 years, literacy, non-pregnant women and living with a spouse for both groups. Specific criteria for fertile women included at least one healthy alive child and woman not in puerperium. Specific criteria for infertile women included no pregnancy after one year of regular, unprotected intercourse, primary infertility and infertility confirmed by a gynecologist. Exclusion criteria included women addicted to narcotics, alcohol and drugs affecting sexual function, stressful event during past one month, certain medical disease, and psychological disorder. If the couples together completed the questionnaires, they were also excluded.

After receiving permission from the Ethics Committee, the researcher attended at the health center or infertility center in the selected days from 8-12 A.M., and selected the qualified people among fertile couples referred to health centers for receiving the services (immunization, family planning, etc.) and infertile couples referred to Mashhad Montaserieh Clinical Research Center for diagnosis, treatment or counseling for infertility measurements who had the inclusion criteria and were satisfied to participate in the study.

In compliance with privacy, each woman is guided to a quiet and peaceful place and they were informed about the study. Subjects were ensured that this information is confidential and will be provided if needed. Then, after obtaining the written consent, demographic information form was completed by interview. After giving necessary information on how to complete the questionnaire; questionnaire was completed individually and simultaneously. To appreciate their cooperation, a training booklet about sexual training was given to them at the end. If there were sexual problems in couples referring to psychologist or doctor was recommended depending on their sexual problem. Data collection tools included interview form consisted of 4 parts: demographic-family characteristics, marital life, questions specific to fertile and infertile couples and Glombok-Rust sexual status questionnaire (GRISS-F1) which consisted of 28 questions to assess the presence and severity of sexual problems (19, 20). 

The questions on a five- degree Likert scale assess the type and severity of sexual problems in seven areas in two separate form for women and men from 0-4 score. Minimum score in this scale was zero and maximum score was 112. Sub-scales of women questionnaire include infrequency, non-communication, dissatisfaction, avoidance, non-sensuality, vaginismus, and anorgasmia. Total score obtained for each subject assesses her/his severity and weakness of sexual problems in a nine-degree continuum, from 1, the lowest sexual problems (in women, score of 0-20, and in men, score of 0-12), to the highest.Golombok-Rust inventory of sexual status-female sexual problems (in women, score >68 and in men, score >50). Validity of the form of demographic characteristics and sexual problems was confirmed with content validity method and reliability of the form of demographic characteristics was confirmed with the method of assessor agreement with a correlation coefficient of r=0.83. 

GRISS questionnaire was designed by Glombok and Rust, and its validity was confirmed with technique of known groups, and its reliability was confirmed with Cronbach's alpha of 0.87 for men and 0.94 for women (15). The validity and reliability of sexual problems questionnaire in Iran were confirmed by Besharat in fertile couples with Cronbach's alpha coefficient 0.84 for women and 0.79 for men, and in infertile couples by Besharat and Hossein Zadeh with a correlation coefficient 0.89 for women and 0.92 for men (10, 26). 

**Statistical analysis**

Data was analyzed using SPSS software (version 16) and descriptive statistics indexes and Mann-Whitney test, Kruskal-Wallis and Pearson correlation tests. In these tests, confidence coefficient 95% and tests power 80% were considered. 

## Results

The results showed that both fertile and infertile groups were matched in terms of all underlying variables. Tables I, II show some of the underlying characteristics of subjects. In this study, 83 cases (75.5%) of fertile and 92 (83.6%) of infertile women were housewives. Mean duration of diagnosis of infertility in infertile couples was 4.85±3.53 yrs, and the mean duration of treatment was 3.62±3.27 yrs. Cause of infertility in 28.2% was female factors and in 40% was male factors, in 10%, combined factor and in 10.9% was unknown. Also, 46 cases (41.8%) had used assisted reproductive techniques. In fertile women, mean of gravidity was 1.56±0.8, and mean number of children was 1.27±0.46. Contraception method in 50 fertile women (45.5%) was withdrawal intercourse.

Mean of sexual infrequency was significantly higher in fertile than infertile women (p=0.002). While no statistically significant difference was observed between two groups in terms of total score and other aspects of sexual problems. Based on nine-degree continuum, fertile and infertile women were at level 3 of sexual problems (score of 26-30). Most sexual problem in infertile women was related to non-relationship and in fertile women was non-frequency (Table III).

There was a significant relationship between the rates of sexual problems in fertile women with menstrual cycle (p=0.015) and lactation (p=0.023), so that sexual problems were less in middle of menstrual cycle and sexual problems were more during menstruation and before it and also in lactating women. Spearman's test results showed a significant inverse relationship between sexual problems of infertile women with education (p=0.003, r=-0.276). There was no significant relationship between other individual characteristics, fertility and marital status with couples' sexual problems.

**Table I:** Demographic data in fertile and infertile couples

**Variables**		**Infertile**		**Fertile**	**Result**	
Female education						
	Basic literacy	0	0		p=0.13, df=1, 2.26=k_2_
	Primary school	1 (0.9)	1 (0.9)		
	Secondary school	8 (3.7)	3 (2.7)		
	High school diploma	54 (49.1)	42 (38.2)		
	College	47 (42.7)	64 (58.2)		
Male education						
	Basic literacy	1 (0.9)		1 (0.9)	p=0.18, df=2, k_2_=3.4	
	Primary school	8 (3.7)		11 (10.0)		
	Secondary school	25 (22.7)		22 (20.0)		
	Diploma	40 (36.4)		39 (35.5)		
	College	36 (32.7)		37 (33.6)		
Residence status						
	Leased	46 (41.8)		58 (52.7)	p=0.19, df=4, k_2_=5.11	
	Owned	42 (38.2)		38 (34.5)		
	Living with husband’s family	17 (15.5)		13(11.8)		
	Living with wife’s family	1 (0.9)		1 (0.9)		
	Other	4 (3.6)		0		
	Total	110 (100.0)		110 (100.0)		

* Chi square test. Data are presented as mean±SD.

**Table II:** Demographic data in fertile and infertile couples

**Variables**	**Infertile**	**Fertile**	**Result** ^**^
Female age (years)	29.2 (4.9)	28.4 (3.9)	p=0.25 / z=1.14
Male age (years)	32.5 (5.1)	32.0 (4.8)	p=0.58 / z=0.56
Female body mass index *	25.2 (4.7)	24.3 (3.9)	p=0.14 / z=1.48
Male body mass index	26.3 (10.6)	25.9 (4.5)	p=0.51 / z=0.67
Duration of marriage (years)	6.9 (3.83)	6.4 (3.9)	p=0.21 / z=1.25

Data are presented as mean±SD.

* Kilograms divided by the square meter

** Mann-Whitney test

**Table III:** The mean and standard deviation of sexual problems in fertile and infertile women

**Sexual problem**	**infertile women**	**fertile women**	* **p-value**
Total	29.0 (15.4)	28.9 (15.5)	0.963
Non-communication	5.5 (2.9)	5.0 (2.8)	0.168
Infrequency	4.7 (2.4)	6.0 (2.9)	0.002
Dissatisfaction	3.3 (3.1)	3.1 (3.2)	0.526
Avoidance	2.9 (3.3)	2.8 (3.2)	0.418
Non-sensuality	3.1 (3.2)	2.4 (2.6)	0.153
Vaginismus	5.0 (3.2)	5.7 (3.5)	0.104
Anorgasmia	4.37 (3.17)	3.87 (3.41)	0.146

* Mann-Whitney test. Data are presented as mean±SD.

## Discussion

In this survey, sexual dysfunction questionnaire in women had six subgroups: infrequency, dissatisfaction, non-communication, avoidance, non-sensuality, vaginismus and anorgasmia. Most sexual disorder in infertile women were related to non-communication and in fertile women were infrequency. There was no significant difference between fertile and infertile women in six subgroups. However most sexual disorders in infertile women were non-communication and in fertile women were infrequency. Sexual infrequency in fertile women was higher than infertile ones.

It can be justified that infertile women are encouraged to lie with a purposeful intercourse. It means every day during middle of month (11, 16, 17). The most sexual dysfunction in infertile women were non-communication which means couple inability to talk about their sexual problem. For couples experiencing infertility, sex begins to resemble a clinical procedure rather than a loving passionate act between husband and wife. Infertility takes the spontaneous out of sex. Attempt to conceive means having sex on right days (18).

In this survey we selected the qualified people among fertile women referred to health centers for receiving the services (immunization, family planning, etc.). Therefore 75% of control group (fertile women) were lactating which had negative impact on sexual function in women. Breastfeeding can cause hypoestrogenic condition, prolactin increasing and testosterone decreasing cause vaginal dryness and low libido.

The most sexual dysfunction in infertile women in other researches were different, for example Tayebi *et al* reported anorgasmia (12), while Jane and Oskay have reported decreased libido (12, 19, 20). The reasons for inconsistency with present study can be using sexual disorder evaluation and questionnaire different tools. We observed based on nine-degree contium, minimum and maximum sexual dysfunction was 1 and 9 respectively. Both of infertile and fertile women had level 3 of sexual problem.

The results of this study showed no significant difference between fertile and infertile women in terms of total score of sexual problems, which is consistent with result of studies of Nelson, Drozdal and Monga (21, 22, 16). Monga evaluated the effect of infertility on quality of life, marital adjustment, and sexual function of infertile couples and reported that women in fertile and infertile groups had no significant difference in terms of sexual problems. Bahrami and Nourani in their studies reported no significant difference between sexual satisfaction in fertile and infertile women (17, 23). Couples with infertility problem think about reproductive aspects of sex rather than enjoying it, so they report less complaints of sexual dysfunction to their clinicians (24).

The study of Besharat and Bazargani fond that infertile women suffer more sexual problems and sexual disfunction are age-related and in women obesity, lower urinary tract symptoms, urinary incontinence and education level seem to have role in sexual dysfunction (16). In addition, another factor to be related with sexual behavior is duration of partnership. We adjusted infertile subjects according to education, residence status, age, body mass index and duration of marriage (25).

Besharat and Bazargan found that infertile women suffer more sexual problem than fertile women, which is inconsistent with our study (10). The reasons for inconsistency with our study may be their low sample size and selecting the subjects among women referred to hospital clinics for treatments that may not be good indicator of fertile women in society.

Oscay also reported infertile women have more sexual disorder than fertiles (20). It may be explained by using different tools, female sexual function index, different duration of infertility culture, attitude and life style. In Iranian society, infertile women are more vulnerable than infertile men, this leads to denying infertility and sexual problem. Thereby, increase psychological adaptation and compensatory mechanisms (26). 

This study was conducted in infertile women with at least 4 years infertility and 3 years infertility treatment. As the infertility duration extends, all dysfunction sexual disorders were increased. Leah *et al* investigated FSFI in 119 infertile and 99 healthy women between age 18-45 years. They showed sexual disorder in 25% of fertile subjects. The score of desire and arousal parameters was significantly lower in infertile group (6). Marital life in Iranian culture and its concepts have some difficulties for women because there is no appropriate language and word to express their sexuality throughtout marriage (27). Merghati *et al* found that Iranians believe talking about their marital lives in public will disrespect them socially (2). 

One of the features of this study was evaluation of sexual problems in both genders and comparison of results with same group of fertile one. We reported in this paper data of women. One of the limitations of this study include cultural issues such as shame for stating sexual problems that can be effective on response of subjects. Also, stress and concerns related to counseling, treatment and diagnosis of infertility in infertility clinic may affect the response of infertile couples. We tried to relatively control this limitation by completing the questionnaire when the subjects were ready in terms of mental status.

## Conclusion

In conclusion, there was no significant difference between fertile and infertile women in terms of sexual problems. Both infertile and fertile women at level 3 of sexual problem was observed. Paying attention to sexual aspects of infertility and presence of programs for training of sexual skills seems necessary for couples. We need to develop practical strategies to improve Iranian couples's awareness in sexuality issues.
